# Neurocutaneous syndromes

**DOI:** 10.1097/JS9.0000000000001816

**Published:** 2024-06-13

**Authors:** De-an Qin, Jun-xia Qin

**Affiliations:** a Department of Spinal Surgery, Shanxi Provincial People’s Hospital; b Department of Dermatology, Shanxi Provincial People’s Hospital, Taiyuan, Shanxi, People’s Republic of China

Highlights

- Neurofibromatosis (NF) and tuberous sclerosis complex (TSC) are the two most common genetic neurocutaneous syndromes characterized by some specific concomitance of tumors.
- Both are of cancer susceptibility, suggesting cancer predisposition syndromes.
- As a minor diagnostic criterion for TSC, spinal sclerotic bone lesions (SBLs) can be seen in many other pathologies, and awareness of the characteristics of different SBLs is important to avoid delayed diagnosis and misdiagnosis.

Cutaneous manifestations can occasionally be the sentinel sign of an underlying neurological lesion and recognizing the external cutaneous alterations is crucial because it can early hint the internal lesion. The concomitant disorders simultaneously involving the nervous system and the skin are named neurocutaneous syndromes, in which neurofibromatosis (NF) and tuberous sclerosis complex (TSC) are the two most common diseases^1^. The pathogenesis of both is due to the loss of tumor suppression in mitogen-activated protein kinase mammalian target of rapamycin (mTOR) signaling pathways. Both are genetic diseases, but clinical-based diagnoses are characterized by some specific concomitance of tumors. Both are of cancer susceptibility, suggesting cancer predisposition syndromes^2^. As hereditary diseases for both, the current mainstream managements are symptomatic treatments, complications prevention, and life-long follow-up.

NF type 1 (NF-1) accounts for more than 96% of NF cases and is inherited from an autosomal dominant mutation of the NF1 gene. It is clinically characterized by hamartomas and tumors in the skin, eye, central nervous system, and other organs. The cutaneous lesions are characteristic. To diagnose according to the accepted clinical National Institutes of Health criteria, at least two or more of the following conditions must be met: six or more café-au-lait macules; at least one plexiform neurofibroma or two or more neurofibromas; freckles in the inguinal or axillary regions; optical gliomas; two or more Lisch nodules; bony lesions such as pseudoarthrosis or sphenoid dysplasia; and a first-degree relative with the condition^3^.

TSC, the autosomal dominant mutation of the TSC1 or TSC2 gene, is characterized by hamartomatous growths in multiple organs. Genetic testing has a 30% false-negative rate. When the diagnosis of TSC is clinically established, genetic testing for further confirmation is unnecessary. The optimal time for genetic testing is before pregnancy for young adults who are affected or at risk, and the most valuable benefit is being able to help assess the potential risks to offspring in advance. The classic triad (Vogt triad) of facial angiofibroma, seizures, and mental retardation is observed in only 30–40% of TSC patients. Dermatologic manifestations such as multiple ungual fibromas (fleshy nodules under fingernails or toenails) or Shagreen patches (thick leathery, pebbly skin abnormalities) are considered major diagnostic criteria. The radiological appearance of sclerotic bone lesions (SBLs) has been reinstated as a minor diagnostic criterion for TSC in the 2021 international TSC consensus^4^. Two major features or one major feature plus two minor features are required for a definite clinical diagnosis of TSC.

As a minor diagnostic criterion for TSC, the radiological appearance of spinal SBLs can be seen in many other pathologies including genetic diseases, metastatic malignancy, lymphoma, osteopetrosis, Paget’s disease, ankylosing spondylitis, etc. Spinal SBLs can be broadly classified as focal, multifocal, and diffuse based on the distribution. Awareness of the characteristics of different SBLs is important to avoid delayed diagnosis and misdiagnosis^5^. Ankylosing spondylitis is characterized by multifocal anterosuperior endplate sclerosis and squaring vertebrae. The sclerotic phase of spinal Paget’s disease appears with vertebral expansion and cortical thickening, which gives rise to the picture frame sign on radiographs. Metastatic SBLs are with primary malignancy mainly from the prostate and breast and typically appear focal or multifocal. Multifocal SBLs of TSC are located characteristically in the middle and posterior vertebral columns (Fig. 1). Skeletal scintigraphy is helpful in distinguishing TSC from osteoblastic metastases, for the former usually has normal radionuclide uptake, and the latter always demonstrates avid uptake. Knowledge of key clinical and radiological features can help confidently identify these SBLs of TSC as do-not-touch lesions and not prescribe unnecessary testing or even vertebral biopsy.

**Figure 1:**
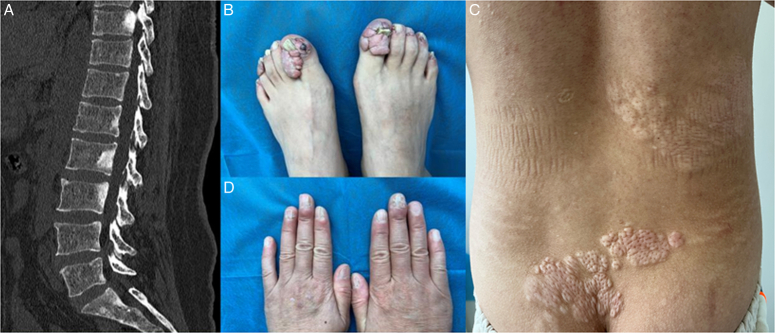
(A) Lumbar sagittal computed tomography reconstruction for a middle-aged woman with tuberous sclerosis complex revealed multiple sclerotic lesions in the middle and posterior vertebral columns. (B, C) Physical examination showed multiple fleshy nodules under fingernails and toenails (ungual fibromas). (D) Her young son had some thick, leathery, pebbly skin abnormalities (Shagreen patches) on the back.

## Ethical approval

We have had the ethics approval and consent of the Shanxi Medical University ethics committee.

## Consent

The case in this article is real, and written consent has been obtained.

## Source of funding

There is no funding for the research.

## Author contribution

All authors have contributed to the planning, data collecting, drafting, and writing the manuscript. De-an Qin is responsible for the revising, final approval, and overall content as guarantor.

## Conflicts of interest disclosure

We have read and understood the policy on declaration of interest and declare no competing financial interests.

## Research registration unique identifying number (UIN)

31286.

## Guarantor

De-an Qin.

## Data availability statement

The data will be made available upon reasonable request.

## Provenance and peer review

Not applicable.
